# Comment on Feuerriegel et al. Assessment of Acute Lesions of the Biceps Pulley in Patients with Traumatic Shoulder Dislocation Using MR Imaging. *Diagnostics* 2022, *12*, 2345

**DOI:** 10.3390/diagnostics13010025

**Published:** 2022-12-22

**Authors:** Frank Martetschläger, Naman Wahal

**Affiliations:** Department of Shoulder and Elbow Surgery, ATOS Clinic Munich, Effnerstrasse 38, 81925 Munich, Germany

We wish to congratulate the authors for the successful publication of the article titled ‘Assessment of Acute Lesions of the Biceps Pulley in Patients with Traumatic Shoulder Dislocation Using MR Imaging’. *Diagnostics* 2022, *12*, 2345 and add some points for consideration [1].

The authors have mentioned the incidence of biceps pulley lesions as lesions of sGHL alone (superior Gleon-humeral Ligament), lesions of CHL alone (Coraco-humeral Ligament), or a combination of both sGHL and CHL. The classification system used is mentioned as the one given by Habermeyer (Table 2, reference no. 8 of the article). However, the classification system described by Habermeyer does not mention lesions of CHL [2]. This is a false representation of the classification system and leads to considerable confusion when reading the article.

To mitigate this problem, we propose using the simpler and more recent classification system of biceps pulley lesions given by Martetschläger et al. [3]. This classification system (Table 1) also considers the lesions that do not involve sGHL (36% of the cases reported in the current article).

## Figures and Tables

**Table 1:** Martetschläger classification of biceps pulley lesions.

Martetschläger Classification of Biceps Pulley Lesions [3]		
Type 1	Lesions of medial sling	sGHL and mCHL
Type 2	Lesions of lateral sling	lCHL
Type 3	Medial and lateral sling lesion	sGHL and CHL

sGHL: superior Glenohumeral Ligament. CHL: Coracohumeral Ligament. mCHL: medial Coracohumeral Ligament. lCHL: lateral Coracohumeral Ligament.
